# Promoting Universal Equitable Accessibility: An Overview on the Impact of Assistive Technology in the UN, UNICEF, and WHO Web Portals

**DOI:** 10.3390/healthcare11212904

**Published:** 2023-11-05

**Authors:** Rossella Simeoni, Antonia Pirrera, Paola Meli, Daniele Giansanti

**Affiliations:** 1 Facoltà di Medicina e Chirurgia, Università Cattolica del Sacro Cuore, Largo Francesco Vito, 1, 00168 Roma, Italy; 2 Centro Nazionale TISP, Istituto Superiore di Sanità, Viale Regina Elena 299, 00161 Roma, Italy

**Keywords:** assistive technology, accessibility, WHO, UNICEF, UN, ISO, need, disability

## Abstract

The number of people with disabilities and frailties who need support and assistance is increasing. Assistive technologies (ATs) are increasingly playing a central role in supporting people with disabilities and frailties. The study investigated the impact of the ATs on the websites of the UN, UNICEF, and WHO in terms of proposed activities and actions. The methodology proposed was based on two points of view: (1) A formal process to directly select elements in the institutional webs of the UN, UNICEF, and WHO. (2) A formal process for a complementary literature narrative review based on an *umbrella review* of Pubmed and Scopus. A standard checklist and a qualification process were applied. The outcome reported 35 documents from the direct search on the web and 19 systematic reviews for the complimentary literature overview. The direct search returned documents related to initiatives focused on the following: The tailoring of the ATs to a person based on international guidelines and specific monitoring initiatives of the AT introduction/access based on surveys both at the population and system/government level with the publication of the data/metadata in an observatory. Dissemination initiatives of both the culture of ATs (e.g., catalog, guidelines, reports, congresses) and of recommendations. The literature overview contributed more specifically to the use and effectiveness of categories of ATs. Both direct research and the literature overview have shown a consistent growth in interest in ATs. The initiatives of the UN, UNICEF, and WHO have been consistent with the institutional role and aimed at improving the diffusion of ATs through capillary monitoring, which is not free from obstacles, and a diffusion of the culture and rational use of ATs. The narrative review shows also the important role of research in monitoring the development, use, and effectiveness of devices, strategies, and support of international institutional initiatives. Important initiatives have been launched internationally on AT in terms of monitoring, dissemination, and improvement in access. However, it is necessary to consider and face the obstacles that limit these initiatives.

## 1. Introduction

### 1.1. Background and Key Questions

The terrific development of information and communication technology (ICT), together with the high technological Assistive Technologies (ATs) [1,2,3], has the potential to improve the quality of life and integration into society of many people with the most diverse disabilities.

In recent years, we have assisted in the following ways:

1. The breadth of technologies on offer. Over the course of a century, technical and technological aids for disabled and frail people have greatly improved, and now the focus and attention is on expanding ATs’ accessibility both from economic/social and technological points of view by providing free supplies.
2. The evolution of technological tools themselves. We have gone from rudimentary mechanical supports, such as the first prostheses and the first wheelchairs, up to modern tools that make extensive use of electronic and information technology components.
3. The perception of society towards aid for disabled and frail people, both from a common point of view and from an institutional point of view. In recent years, for example, the United Nations Convention on the Rights of Persons with Disabilities established that every member state should compulsorily guarantee and offer its citizens access to mobility aids, assistive devices, and assistive technologies for disability.

It must be highlighted that the demand for these ATs is increasing due to the aging of the population, which is leading to a growth in people with disabilities in need of support. The ATs that are being developed are increasingly following the indications of the International Classification of Functioning, Disability and Health (ICF) [4,5,6] that are tailored to an individual.

However, there are disparities in AT access and allocation worldwide both at the level of social categories and at the level of more and less developed countries. It is important to understand how this problem is being addressed internationally and to understand what actions are being implemented. The United Nations (UN), with its main institutional components that deal with health—the World Health Organization (WHO) and the United Nations Children’s Fund (UNICEF)—plays a leading role in this area [7,8,9,10,11,12].

This overview focuses on the UN and its institutional components dedicated to the health domain and analyzes the evolution of the approach towards ATs and the activities implemented in this context with reference to monitoring, dissemination, and recommendations. 

The overview aims to answer the key questions:

*“How has the approach to ATs evolved in these international bodies according to the WEBs?”*

*“What are their monitoring initiatives in this area?*

*“What are their dissemination initiatives both in terms of data and metadata and of contents and information?”*

*“What are the problems and bottlenecks?”*

### 1.2. Organization of the Study

The study is arranged into five sections.

The *first section* is the introduction that presents the problem to be addressed through a review and the questions to be answered and reports the organization of the study

*Section 2, “Methods”,* illustrates the study design, which has two polarities.

The first polarity is reported in *Section 2.1, “Web Search in Detail”,* describing the search method in the institutional sites of the UN, UNICEF, and WHO, including the formal process based on an algorithm. *Section 2.2, “The Narrative Review Designed as an Umbrella Review”,* reports the methodology carried out in an umbrella review around these themes, including the formal process based on a checklist and a qualification procedure.

*Section 3, “Results”,* reports the outputs of these two searches.

*Section 3.1, “The Evolution of the Inclusion of the Assistive Technologies in the Websites”,* traces the evolution of the frequency of occurrence of the term AT in the three sites over time.

*Section 3.2 “The Outcome of the Web Search”,* is dedicated to the output of the search on websites. It has been organized into four themes structured editorially in the following four subparagraphs:

- *3.2.1 Role and Mandate of the United Nations, UNICEF, and the WHO on Assistive Technologies*
- *3.2.2 The Focus on the Individual on the Basis of the ICF*
- *3.2.3 Monitoring and Dissemination Initiatives*
- *3.2.4 Recommendations*

*Section 3.3 “The Outcome of the Narrative Review Based on the Umbrella Review”* reports the outcome of the umbrella review organized into the following themes structured editorially in five subparagraphs:

- *3.3.1 Exploring the Interconnection between Assistive Technology and the Environment.*
- *3.3.2 The Crucial Role of Internet and Electronics in Enhancing Assistive Technology.*
- *3.3.3 Empowering Motor Disabilities through Assistive Technology Solutions.*
- *3.3.4 Enhancing Independence for Individuals with Cognitive and Communication Disabilities through Assistive Technology.*
- *3.3.5 Addressing the Accessibility Gap: Improving Access to Assistive Technologies in Low-Income and Middle-Income Countries.*

*Section 4* is dedicated to discussion and is organized into two discussion topics. The first topic, reported in *Section 4.1, “Interpretation of results”*, addresses and interprets, on the one hand, the evidence of the direct research on the websites (*Section 4.1.1 Direct Evidence from the UN, UNICEF, and WHO Websites*), and on the other hand, it discusses the contribution of the umbrella review to the general analysis and to a more complete interpretation of the first research (*Section 4.1.2 ”The Contribution of the Umbrella Review”*). The second topic in *Section 4.2, “Final Thought: The Bottlenecks”,* reports the bottlenecks that emerged.

*Section 5* draws the conclusions of the study. 

## 2. Methods

This review used a standardized checklist designed for the narrative category of reviews (see at https://it.scribd.com/document/434616519/ANDJ-Narrative-Review-Checklist (accessed on 6 September 2023)).

The literature review was essentially based on web searches of institutional sites. Furthermore, to verify whether an added value could be obtained from international publications compared to direct searches on the webs of international institutions, a narrative review designed as an *umbrella review* (which considers the produced systematic reviews) was performed based on targeted searches using specific composite keys on Pubmed and Scopus. This double approach has also the following rationale.

From one side, it is useful in this emerging field to interrogate the ‘grey literature’, specifically websites, to look at actions related to ATs from global bodies. This could offer the evidence base and be useful to the actors in the health domain to spread findings divided into role and mandate and monitoring and dissemination initiatives. However, this gives a non-exhaustive and complete picture compared with the peer-reviewed evidence base if not supported enough by the peer-reviewed literature. The complementary narrative review may also serve to validate the findings when confirming them and/or to suggest attentiveness when this confirmation is not found because, for example, the theme was not studied in depth by scholars. 

This research ended on 30 June 2023.

### 2.1. Web Search in Detail

The search focused on institutional sites to analyze how the issue of assistive technologies is addressed. The search applied targeted searches on the Institutional websites of the United Nations, UNICEF, and WHO, selecting documents according to a formal process based on a specific algorithm. Information technology provides tools to achieve this. 

It was therefore decided to use the Google search suite, which has its own presentation selection and sorting tools based on an algorithm that uses an automatic ranking based on five parameters: *meaning, relevance, quality, usability, and context* [13]. An algorithm was proposed which foresees two phases. The first selection phase was based on Google ranking (N.1–N.6), and the second phase of further synthesis and refinement (N.7–N.11) of the research included: (a) Selection based on our parameters evaluated by means of a graded score ranging from 1 = minimum to 5 = max. These parameters have been identified as *priority, pertinence, completeness, and effectiveness* of the transmitted message. Only items with all 4 parameters with a rating higher than 3 (in AND logic) were taken into consideration. (b) An overlapping check between the various documents followed by a further synthesis operation. 

#### Algorithm Used in the Web Search

1. *Start* by selecting the websites *un.org*, *who.int*, and *unicef.org*.
2. Perform three searches using the following queries:

*2.a-> site: unicef.org assistive technology*

*2.b-> site: un.org assistive technology*

*2.c-> site: who.int assistive technology*

3. Collect the results from the first 10 pages of each search, ordered by the priorities of the *Google ranking (meaning, relevance, quality, usability, and context)*
4. *Exclude the commercial content.*
5. *Analyze the chronological evolution* of the results within each site, with a focus on the entire history, the last ten years, and the last three years (pandemic period) ordered according to the Google ranking.
6. *Select documents* with a focus on assistive technology, including web publications and documents that can be reached by means of ramifications starting from these websites.
7. *Assign a personal score from 1 (min) to 5 (max)* to each selected document based on the following parameters:

*Priority;*

*Pertinence;*

*Completeness;*

*Effectiveness*.

8. Select only those documents that exceed a threshold of three on all parameters defined in step 7.
9. *Check for overlaps* among the selected documents and *select those with higher scores*.
10. *Carry out a synthesis* of the selected documents.

### 2.2. The Narrative Review Designed as an Umbrella Review

The literature overview accompanying the main survey was conducted using both a qualification checklist and a qualification methodology based on the proposed quality parameters described in [14] used to decide the inclusion of the study in the overview.

#### 2.2.1. Algorithm Used in the Literature Overview

1. Set the search query to:

“defined search query”

2. Conduct a targeted search on Pubmed and Scopus using the search query from step 1.
3. Select studies published in peer-reviewed journals that focus on the field.
4. For each study, evaluate the following parameters:
  - N1: Is the rationale for the study in the introduction clear?
  - N2: Is the design of the work appropriate?
  - N3: Are the methods described clearly?
  - N4: Are the results presented clearly?
  - N5: Are the conclusions based and justified by the results?
  - N6: Did the authors disclose all their conflicts of interest?
5. Assign a graded score to parameters N1-N5, ranging from 1 (minimum) to 5 (maximum).
6. For parameter N6, assign a binary assessment of “Yes” or “No” to indicate if the authors disclosed all their conflicts of interest.
7. Preselect studies that meet the following criteria:
  - Parameter N6 must be “Yes”.
  - Parameters N1-N5 must have a score greater than 3.
8. Include the preselected studies in the overview.

## 3. Results

### 3.1. The Evolution of the Inclusion of the Assistive Technologies in the Websites

**Figure 1:**
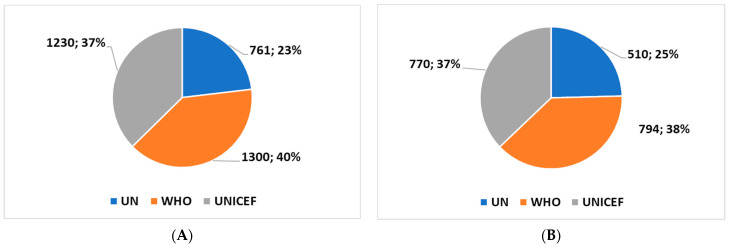
History of citations on “assistive technology” on the UN, UNICEF, and WHO websites in the last 10 years (**A**) and after the outbreak of the pandemic (**B**).

**Figure 2:**
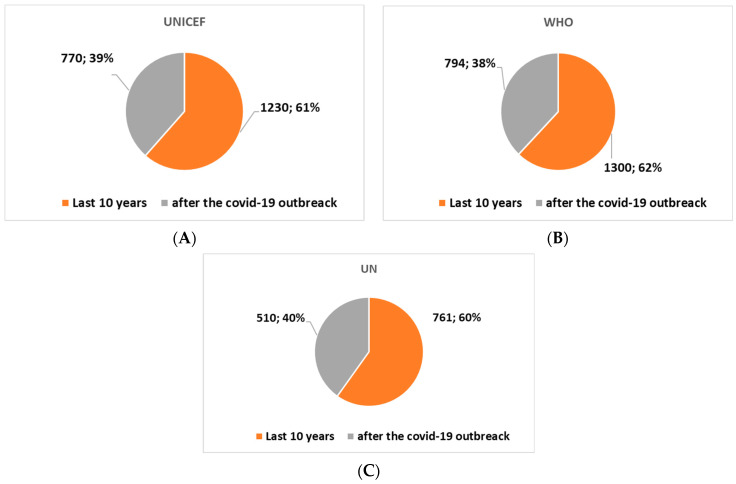
History of assistive technology citations for each separate site over the past 10 years and since the outbreak of the pandemic. (**A**) WHO website. (**B**) UNICEF website. (**C**) UN website.

**Figure 3:**
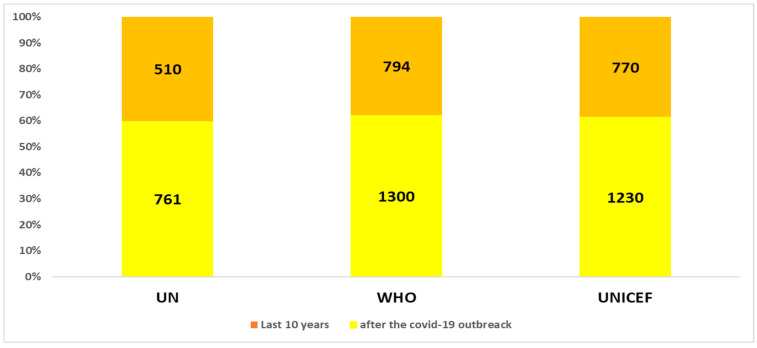
Historical detail for each site in percentage with reference.

The application of the algorithm highlighted the trends in the three websites un.org, who.it, and unicef.org. First, the analysis showed that the first citations were as follows: UN site, on 15 October 1998. WHO site, on 15 October 1992. UNICEF site, on 16 June 1995. Figure 1, Figure 2 and Figure 3 show the trends in the last ten and three years. The analysis shows that (Figure 1), even if not predominantly, the major citations are recorded on the WHO website.

In general, if we consider the last ten and three years (Figure 2 and Figure 3), the analysis shows an increase in the citations of all the institutions’ websites in the period following the outbreak of the COVID-19 pandemic (the last three years). In fact, there is an acceleration in interest over the past three years, presumably due to the push of technological innovation due to the COVID-19 pandemic. In fact, Figure 2 and Figure 3 show that in the last three years, there has been an increase in citations of all three websites. The number of citations has increased by around 40% compared to the ten-year period.

In summary:

- In the last ten years, the term “assistive technology” was cited 1300, 1230, and 761 times, respectively, on the WHO, UNICEF, and UN website.
- After the COVID-19 pandemic outbreak, the term “assistive technology” was cited 794, 770, and 510 times, respectively, on the WHO, UNICEF, and UN website.

### 3.2. The Outcome of the Web Search

The analysis showed that from time to time, initiatives were activated that absorbed the previous ones. Therefore, an important and strategic parameter was certainly that of priority. The defined algorithm search returned 35 documents on the WHO, UNICEF, and UN websites [3,4,6,7,8,9,10,11,12,15,16,17,18,19,20,21,22,23,24,25,26,27,28,29,30,31,32,33,34,35,36,37,38,39,40,41,42].

The analysis, also considering the aims and sub-aims, suggests a division according to the following themes:

○ *Role and mandate of international institutions in assistive technologies;*
○ *The focus on the individual on the basis of the ICF;*
○ *Monitoring and dissemination initiatives;*
○ *Recommendations.*

#### 3.2.1. The Role and Mandate of the United Nations, UNICEF, and the WHO on Assistive Technologies

Research on ATs clearly redirects to the role of international actors operating in this area: the UN, UNICEF, and WHO. The application of the algorithm in *Section 2.2.1* is also traced back to the system information relating to these institutions. It was considered essential to bring them back as they clarify the role and mandate of these institutions in relation to assistive technologies.

The United Nations (UN) was founded on 24 October 1945. Today, virtually every nation on the planet is part of the UN—193 countries in total [7,8]—and must follow the UN Charter. According to the provisions of the UN Charter (see at https://www.un.org/en/about-us/un-charter/full-text (accessed on 30 June 2023)), the United Nations has four purposes reported in Art. 1 that can be briefly summarized as follows:

- *To maintain international peace and security.*
- *To develop friendly relations among nations.*
- *To cooperate in solving international problems and in promoting respect for human rights.*
- *To be a center for harmonization of the different national initiatives.*

The role of the UN in the *health domain* (and therefore around the ATs) is touched upon by point (3) and by point (4). In fact, the right to health is an integral part of the internationally recognized fundamental human rights, as mentioned in point (3). Health is also an undisputed part of the various national initiatives, which the UN has the task of harmonizing, as referred to in point (4). For this task, the UN makes use of two international institutional entities: the WHO and UNICEF. 

The World Health Organization (WHO) [9,10] serves as the primary guiding and harmonizing institution for health at an international level. The WHO’s overarching goal is to ensure that all individuals achieve the highest possible level of health. As per the WHO constitution, health is characterized by complete physical, mental, and social well-being, transcending the mere absence of disease or infirmity, with assistive technologies (ATs) playing a pivotal role.

The United Nations Children’s Fund (UNICEF) [11,12] was initially proposed after World War II to support mothers and children with food and healthcare provisions in nations suffering from devastation. The UN enlarged UNICEF’s role in 1950 in all the nations to face long-term needs. AT is clearly a long-term need. 

#### 3.2.2. The Focus on the Individual Based on the ICF

ATs are those devices or tools capable of improving the functional abilities of people with disabilities, including hardware, software, and mechanical devices. These can support people who have both communication difficulties, for example, hearing, seeing, feeling, memorizing, pointing, and typing, and difficulties with movement. They can be used in domestic environments, in social life environments, and in the workplace. ATs can have high technological components regarding mechanics, information technology, and electronics. According to the WHO, an AT can be defined as both a product and a service [3]. Therefore, according to the WHO, the definition of AT can be extended to the service. The International Organization for Standardization (ISO), which strictly collaborates with the WHO in the WHO Family of International Classifications Network, gives an exhaustive definition of an assistive product (https://www.iso.org/standard/72464.html (accessed on 4 october 2023)), clearly identifying the inclusion. Assistive products, according to the ISO classification, are also those products utilized by an individual to enhance functionality and minimize disability, yet necessitating assistance from another individual for their operation, and are encompassed within the classification. The following items, for example, are expressly not covered by the classification: items utilized for installing assistive products; solutions resulting from combinations of assistive products that are classified individually in the ISO document; medications; assistive products and tools exclusively used by professionals in the health domain or professors; guide dogs; financial support; implanted devices. An important aspect related to AT is the correct assignment to a person with need. These needs are identified by means of a careful analysis of the health components of the person. A globally consolidated guide to identifying the components of health has been identified in the International Classification of Functioning, Disability and Health (ICF) [4,5,6]. Evolving from the earlier International Classification of Impairments, Disabilities, and Handicaps (ICIDH) established by WHO in 1980 for research purposes [15], the ICF signifies a major revision. The ICF’s final text received approval during the 54th *World Health Assembly* (WHA) on 22 May 2001. Subsequently, it was highly recommended to member states for extensive utilization in diverse domains such as research, population studies, and reporting. The classification has also been activated by the WHO based on the actors involved [16].

The ICF (a) allows you to identify the health components of an individual—it has universal use and does not only concern people with disabilities, but everyone can be associated with one or more qualifiers related to “functioning”; (b) takes into consideration the entire span of life; and (c) takes into consideration the environment and social life surrounding an individual. From the point of view of assigning an AT, the ICF leads to greater attention on the individual and to tailoring of the device, which must consider the state of all health components.

Recently, some international initiatives are trying to make the ICF tool more useful. The AT Service Method (ATSM) seems promising for this. It is an evidence-based process framework to efficiently manage the several parameters required for the efficient provision of an AT. The ATSM is based just on the ICF to establish a universal and internationally appropriate, “person-centred, cross-disability, interdisciplinary, trans-environmental methodology supporting data collection to inform practice and policy on AT interventions” [17]. However, the ATSM has not received an evident interest from scholars in scientific publications. 

A second promising initiative at the international level is linking the Global Burden of Disease (GBD) [18]. to the ICF and ISO 9999 

Diseases are classified according to the International Statistical Classification of Diseases and Related Health Problems (ICD-10). The ICD-10 is the international standard diagnostic classification for all general epidemiological purposes, many health management purposes, and clinical use [19]. GBD is based on the ICD-10. ICF practically complements the ICD-10 but does not contain the functional status in the classification. ATs used to improve a person’s functioning are classified by ISO 9999.1. It is a classification at an international level of ATs. So, this action by the WHO is strategic to put the person at the center by linking the key strategic documents ICD-10, ICF, and ISO 9999. 

#### 3.2.3. Monitoring and Dissemination Initiatives

The development of articulated and structured initiatives based on different levels is recorded at an international level. Monitoring actions based on specific surveys are intended for both citizens and various actors and are combined with uploading actions and dissemination of structured data and metadata on portals visible to all at an international level. Of course, we are witnessing the dissemination of guidelines, book catalogs and congress types. News aimed at achieving specific objectives also falls within this ambit. According to the UN, disability can increase poverty and widen social inequalities between countries. ATs are directly included in the sustainable goals of the UN (SDG 1: no poverty, SDG 3: good health and well-being) [20,21]. To achieve these objectives, it is necessary to pay attention to the most defenseless populations in the world, including people with disabilities. According to the UN, there are some factors that prevent the osmosis of ATs between rich and poor countries [22]. Among these factors, the most important is that technological innovation has focused on highly technological products that are marketed at high cost in rich countries and do not meet the needs of people with disabilities in poor countries.

We must record that the UN also gives specific indications on the use of high-tech ATs to its collaborators and guests with disabilities at events. The UN supply model in this case is described in [23,24], where it is highlighted how accessibility is merely guaranteed through high-tech devices.

Basically, on the one hand, the UN suggests the types of high-tech ATs (HT-AT) as part of its institutional activities but recognizes that these ATs, at least in part, are not suitable for use in the field in less developed countries and communities, where both costs and environmental conditions have to be taken into account. The message that clearly appears in the news shared in the grey literature is that it would be necessary to overturn this paradigm and develop projects aimed at providing a low-tech alternative AT (LTA-AT), adaptable to the environments of these countries and more convenient than the traditional devices available. This point is very important and challenging to address. Specific studies should help us answer how to address this issue, which, in a certain sense, interrogates the usefulness of LT-ATs versus HT-ATs in low- and middle-income countries. International initiatives, such as AT2030 (described below), should, as highlighted in the message, give space to these themes.

The WHO and UNICEF have the prerogative to be engaged in monitoring initiatives in this field. The WHO plays a leading role in monitoring both the needs and diffusion of ATs and the policies adopted for their usability within countries, addressing/guiding the national policies towards a better assignment and diffusion of ATs. UNICEF, which focuses on children and everything that revolves around them, including motherhood, has an important role in monitoring their needs, even in the medium and long term, and ATs represent an indispensable need for many children. *It can be affirmed that the WHO and UNICEF operate in complementarity and synergy.*

The WHO identified five major challenges for the ATs at the international level, as reported in the online documents [1,3,10]:

- *The policy*. It must be considered that in many countries, a policy on ATs is not available.
- *The products*. More focus on the person is needed. Based on the ICF [4,5], every individual is unique.
- *The provision*. The process of provision of an AT must be more effective. Often, these services are not optimized and citizens must follow several often-unconnected passages in order to obtain the right AT.
- *The personnel*. The training of the personnel is also a key issue to ensure both a correct prescription and a well-organized follow-up.
- *Assistive technology within universal health coverage*. This is the most ambitious goal of the WHO. Everyone must access both products and services with no restrictions from an economic point of view.

In line with these challenges, the WHO has designed and developed a global health observatory [25]. A section of this observatory is dedicated to the ATs [26].

A specific WHA resolution (WHA71.8) [27] is focused on ATs; on the one hand, it pushed all countries to improve access to ATs, and on the other hand, it asked the WHO to monitor and collect scientific evidence in the form of a global report. This resolution calls for such reports to be issued in the years 2022, 2026, and 2030 to monitor progress in this area. In response to these requests, the WHO has acted and taken steps to gather this evidence. Data are collected using two main tools based on specific questionnaires designed by WHO [26]:

- The *first tool* is the rapid Assistive Technology Assessment tool, which runs at the level of the population to obtain population data on access to ATs ( https://www.who.int/publications/i/item/WHO-MHP-HPS-ATM-2021.1 (accessed on 6 September 2023)) [28]. It is a questionnaire focused on the population designed to assess, based on a self-reporting methodology, the perceived barriers to access to an AT, the need, the use, and the met need.
- The *second tool* is a questionnaire for obtaining the progress indicators for access to assistive technology that runs at a system level to obtain *specific indicators on the state and advancement of the ATs* (https://www.who.int/publications/i/item/WHO-MHP-HPS-ATM-2022.01 (accessed on 6 September 2023)) [29]. It is a questionnaire allowing to measure, with reference to an AT, the preparedness in terms of several issues, such as the service provision attention, the workforce obtainability, the training governance, the regulation, the national budget, the funding mechanisms, the standards, the projects, and the activities.

Data are stored and available in two windows of the observatory [26]. They are available publicly and separated into two related collections. The first collection is related to the survey at the system level (progress indicators). The second collection is related to the population survey. The data and metadata available in the observatory show that 70 countries provided progress indicators, while 29 furnished the results of the questionnaire. Key global results available on the observatory are as follows: 2.5 billion people are in need of ATs. It is expected that this number will increase to 3.5 billion in 2050. Two-thirds of people with an age ≥ 60 need an AT. Among the 70 countries providing progress indicators, 62 have specific legislation on ATs. In total, 63 countries have measures designed to cover users’ AT costs partly or fully.

As a further initiative, the WHO is developing another tool (not yet available) [30]. It is a population-based questionnaire aimed to assess the impact of an AT on citizens. It will assess the enjoyment of human rights and the people’s empowerment/dignity/participation/quality of life/inclusion.

The WHO also provides *scientific dissemination of data and metadata as per outcomes from the administration of these questionnaires*. The data include, but are not limited to, the following: data on the availability of government/registered services for assistive technology, which may be viewed online [31]; data on the existence of regulations/standards/guidelines/protocols on assistive technology [32]; data on the availability of education/training for assistive technology [33].

Another level of scientific dissemination by the WHO consists of specialized volumes on specific issues related to ATs.

Three have been identified (all accessible online) by relevance, priority, interoperability, and functional interconnection. Together they address the subject of priority ATs at reasonable prices, process and development specifications, and third-party supply through tenders.

The first volume reports and describes those ATs that are highly needed and are fundamental to preserving or increasing an individual’s functioning and, very importantly, need to be available at an affordable price for the community/country [34].

The list of these ATs is called the World Health Organization (WHO) Priority Assistive Products List (WHO APL). These ATs cover six key areas of functional difficulties: mobility, hearing, vision, cognition, communication, and self-care.

The second volume is dedicated to ATs’ specifications [35]. In detail, it reports the model specifications as a reference for the procurement by working groups. These specifications support these groups in the design and development of their own procurement specifications suitable for their settings. In total, 26 AT specifications are included. These ATs are those included in the WHO APL [34]. Particular care is dedicated to the description of the minimum requirements related to both the technological performance and functionality that an AT in the list must meet for safety and effectiveness of use.

The third volume is dedicated to public procurement, precisely to proffering ATs, accessories, auxiliary components, and connected services [36]. The document aims to:

- Overview the procurement and supply management for ATs.
- Describe essential principles of AT procurement.
- Categorize the diverse participants related to the procurement process.
- Detail the phases of the AT procurement process.
- Report other ways of obtaining ATs (e.g., donations of used ATs).

The WHO also proposes itself as an observatory of the dissemination experiences of the various member states of the UN. From this point of view, it represents a real catalog of shared experiences in the field of ATs. See, by way of a non-exhaustive example, the experience in Buthan shared in [37]. UNICEF [38] is working on ATs with objectives aligned with those of the WHO with particular reference to children, mothers, and related living environments. It is also dedicated to the advocacy of ATs. Regarding this aspect, UNICEF fought for the introduction of 24 new AT products into the catalog, including specific models of wheelchairs for children and innovative hearing aids [39]. Thanks to this initiative, UNICEF can distribute innovative acoustic and motor support devices both in international development projects and to various governments of the United Nations. Together with the WHO, it has focused on the development of guidelines for the actors in the AT supply process. Especially important in this direction is a book that represents a guide dedicated to 26 priority AT products, which also contains the specifications and requirements of the production in quality [35]. Also worth mentioning is a global initiative, called AT2030 [40], that works to improve access to life-changing assistive technology (AT) for all. In this context, workshops were held in South Africa and Tajikistan at the end of 2019, and a webinar on ATs was held in September 2020 [41] in collaboration with the WHO and the Clinton Health Access Initiative. 

#### 3.2.4. Recommendations

UNICEF and the WHO developed the “The Global Report on Assistive Technology” in synergy [42], addressing the WHA 71.8 resolution in May 2018 [27] on the international improvement of access to ATs. 

The report on ATs gives a complete vision of international access to ATs, with a focus on several issues, including the need and the expected economic benefits.

It is clearly a disseminative product. However, most importantly, it is dedicated to providing recommendations. The key message of each recommendation (see [42] for the extended text) is reported in Table 1. 

### 3.3. The Outcome of the Narrative Review Based on the Umbrella Review

A search of scientific publications was carried out to complement the overview carried out on the web. It was based on an *umbrella review*.

The composite key in Box 1 was used.

The application of this key led to the identification of 675 published works on Pubmed with a starting date of 1990.

An analysis of the last 10 years shows a number of papers equal to 510 (76%). Instead, if we consider the last three years characterized by the COVID-19 pandemic, we find 238 papers (35.3%). This highlights a rapid acceleration in publications in this area over the past decade. This acceleration becomes terrifying if we consider the last three years characterized by the COVID-19 pandemic. In fact, more than a third of the papers published in this area have been published in the last three years.

In line with the objectives of the study, the investigation focused on the publications in the systematic review category in order to provide an *umbrella review* of the systematic reviews, which, in evidence-based medicine, have a higher hierarchy level and are the first step at the national and international level for the formation of guidelines and recommendations. 

The research reported 19 systematic reviews [43,44,45,46,47,48,49,50,51,52,53,54,55,56,57,58,59,60,61]. Almost all the reviews dealt with aspects of effectiveness and/or acceptance in some way, and several also dealt with multiple issues at the same time. It should be noted that only one review [49] addressed the issue of disparity in the assignment and use of ATs. The following issues emerge from the analysis.

- *Exploring the interconnection between assistive technology and the environment.*
- *The crucial role of the internet and electronics in enhancing assistive technology.*
- *Empowering motor disabilities through assistive technology solutions.*
- *Enhancing the independence of individuals with cognitive and communication disabilities through assistive technology.*
- *Addressing the accessibility gap: improving access to assistive technologies in low-income and middle-income countries.*

Box 1 **The proposed composite key.**

“assistive technology”[Title/Abstract] AND (“UN”[Title/Abstract] OR “WHO”[Title/Abstract] OR “UNICEF”[Title/Abstract])

#### 3.3.1. Exploring the Interconnection between Assistive Technology and the Environment

Three studies [43,48,57] highlight the significance of the environment in the International Classification of Functioning, Disability and Health (ICF) as a crucial factor for protection and participation. In the study referenced as [43], the effectiveness of environmental interventions in preventing falls among older adults living independently was examined. Home fall hazard reduction interventions were shown to notably decrease overall fall rates by 26% in older individuals. However, interventions targeting improved vision or other assistive technologies did not exhibit a substantial impact on fall rates. The review emphasized tailoring home fall hazard reduction strategies for those at higher fall risk, underlining robust evidence supporting their efficacy. Yet, further investigation is needed for other intervention types and population groups. The overview presented in [48] evaluated rehabilitation interventions for middle-aged individuals with long-term physical disabilities. The interventions addressed various aspects such as fall risk reduction, functional capacity, and community mobility. Wheelchair skill training programs (WSTPs) for manual wheelchair users and post-stroke limb exercises were strongly supported by evidence. These interventions should be routinely offered to this population, with calls for tailored interventions due to the unique challenges they face. The review presented in [57] aimed to gauge the impact of occupational therapy on post-stroke adults’ abilities to perform daily activities. Occupational therapy targeting activities of daily living (ADL) demonstrated enhancements in performance scores, decreased risk of poor outcomes, and improved extended ADL independence. However, the evidence’s quality was compromised by methodological flaws and data gaps. While suggesting potential benefits, the review emphasized the necessity for more rigorous research in this domain.

#### 3.3.2. The Crucial Role of Internet and Electronics in Enhancing Assistive Technology

Five systematic reviews [44,45,53,55,60] emphasize the significance of the interplay between electronics, the internet, and assistive technologies (ATs).

The review in [44] delves into the creation of an internet-based intervention for remote monitoring and support of older adults and caregivers using assistive technology. Through an evidence-based, user-centered, and pragmatic approach, the study demonstrates the complexities of crafting such interventions. By combining intervention mapping and participatory processes, the development team established a link between evidence, user needs, and practicality. This work serves as a guide for future developers, shedding light on challenges and considerations. In [45], the research explores the experiences of older Western adults with chronic illnesses and their families using digital technology for aging in place. The study presents a conceptual model depicting how these individuals reflect and decide on using digital devices. Understanding these experiences informs assistive technology development and provides guidance for healthcare professionals supporting older adults and their families. A specific review [53] investigates electronic assistive technology (EAT) effects on the well-being of older adults living alone. EAT displays potential for enhancing physical and mental well-being, though social well-being evidence is limited. Personalized designs, interventions, and user engagement are recommended for EAT design. More evidence is necessary for a comprehensive understanding.

The overview [55] examines ATs and internet access technologies for the deafblind. Early-stage technologies show promise, yet their efficacy remains untested. Bridging the gap between research and real-world implementation is crucial, requiring user involvement and focused development to cater to the unique needs of the deafblind population.

Lastly, [60] focuses on EAT effects on reading, educational outcomes, and quality of life for visually impaired children and young individuals. Despite an extensive search, no relevant randomized controlled trials were found. The study calls for high-quality evidence to assess EAT’s utility for visually impaired children, suggesting that research protocols should consider various outcomes relevant to families and educators.

#### 3.3.3. Empowering Motor Disabilities through Assistive Technology Solutions

Two studies [46,50] specifically addressed the empowerment of people with motor disabilities using assistive technology solutions. However, the studies reported in *Section 3.3.1* [43,48,57] also touched on this field, although to a lesser extent and from a different angle. 

In the study detailed in [50], the impact of motor neuroprosthesis (MN) on post-stroke individuals’ independence in activities of daily living (ADL), limb-related activities, health-related quality of life (HRQoL), exercise capacity, balance, and adverse events was examined. The evidence indicated that MN did not provide superior benefits compared to other assistive technology devices for improving limb-related activities like walking speed, balance, exercise capacity, and HRQoL. Insufficient evidence existed to assess MN’s effect on ADL independence. The study found that MN did not increase fall risk or serious adverse events, but dropout rates during the intervention period were potentially higher. The overall certainty of evidence ranged from low to moderate.

The review discussed in [46] analyzed wheelchair design, particularly in terms of navigating stairs and flat surfaces. It revealed a gap in meeting wheelchair users’ functional needs, as the existing literature primarily concentrated on stair-climbing ability while neglecting safety, comfort, and maneuverability. The review stressed the importance of designing assistive technologies like wheelchairs with empathy, addressing users’ physical and emotional requirements. Overall, it underscored the necessity for comprehensive wheelchair design beyond stair climbing, encompassing a wide array of user needs. By prioritizing safety, comfort, and user-friendly features, designers can create assistive technologies that offer optimal accessibility and functionality.

#### 3.3.4. Enhancing Independence for Individuals with Cognitive and Communication Disabilities through Assistive Technology

Most of the systematic reviews, seven in total [47,49,52,54,56,58,61], dealt specifically with AT in cognitive and communication disabilities. In addition to these works, others, reported in *Section 3.3.2* [44,45,53,55,60], have addressed the importance of the internet and electronics in TA and touched on this topic.

In [47], a study assessed the efficacy of technology aiding braille literacy education for blind or visually impaired children and youth. Despite braille’s importance, limited research on technology for braille literacy education exists. The study emphasized the need for technology evaluation standards, promoting real-time feedback, independent study, editing, user-friendliness, and engagement. Developing such a technology and consistent evaluation standards could significantly impact the rehabilitation and education of visually impaired youth. The review described in [49] aimed to identify variables influencing AT use among individuals with deafness and blindness, contextualized within the International ICF framework. Usability challenges emerged, especially for devices relying on non-visual and non-auditory senses. The lack of research on haptic and tactile aids highlighted the urgent need for technology development catering to this marginalized population. An overview [52], based on a structured review protocol [51], investigated the experiences of carers of dementia patients using ATs. Positive and negative findings were reported across various ATs, emphasizing the importance of a standardized classification system and a family/carer-centered approach in future research. Another overview [54] focused on ATs for tinnitus care, using mixed methods. The study incorporated evidence of efficacy and patient priorities, aiming to facilitate shared decision-making between clinicians and patients and meeting quality standards for decision aids. The review detailed in [56] explored haptic sensory substitution technologies for individuals with sensory disabilities. Such technologies showed potential in alleviating language, communication, and navigation deficits, though acceptance issues persisted. The study highlighted the need for miniaturized, custom-designed, and affordable haptic interfaces to integrate with personal devices like smartphones. In [58], an overview examined robots for rehabilitating and educating children with cerebral palsy (CP) and autism spectrum disorder (ASD). The study called for more valid research and a user-centered design approach to develop affordable robots for these populations, suggesting interdisciplinary involvement and low-cost robotic systems. Finally, the study presented in [61] conducted a systematic review of assistive technology for cognition (ATC) interventions in clinical populations. It categorized cognitive domains and tasks supported by ATC, finding effectiveness in various cognitive functions. The study contributed a framework for ATC prescription based on cognitive deficits, introduced a novel ATC classification, and identified future research and development directions.

#### 3.3.5. Addressing the Accessibility Gap: Improving Access to Assistive Technologies in Low-Income and Middle-Income Countries

Only the study detailed in [59] directly addressed the issue of limited access to assistive technologies (AT) in low-income and middle-income countries (LMIC), particularly concerning aging populations. The researchers conducted a literature search across six countries—Brazil, Cambodia, Egypt, India, Turkey, and Zimbabwe—all of which had ratified the UN Convention on the Rights of Persons with Disabilities and were anticipated to experience rapid growth in the 65 and above population. The findings demonstrated that while these countries possessed AT solutions tailored for older adults with existing impairments and disabilities, there was a notable lack of AT options focused on preventing impairments and disabilities among older adults without current impairments. The study underscored the urgency of a comprehensive, integrated approach within the health and social systems of LMIC to enhance AT availability for aging populations. This approach should encompass promoting low-cost AT initiatives, increasing awareness and AT capacity, bridging the gap between AT policy and practice, and promoting targeted research on AT.

## 4. Discussion

### 4.1. Interpretation of Results

#### 4.1.1. Direct Evidence from the UN, UNICEF, and WHO Websites

We are witnessing both population growth and gradual aging. This is leading to the growth in people with disabilities who need support and assistance.

ATs will increasingly assume a central role [1,2,3,62] in this area and will have to be increasingly centered on the needs of people and their living environment. In fact, from the analysis of the scientific dissemination of ATs in international sites in the last three years (connected with the explosion of the pandemic), there has been a growth in interest in these devices, which, in the critical phases of the pandemic, proved fundamental. In general, it can be observed, on the one hand, that thanks to the actions of these international bodies, the ICF is increasingly guiding us towards a greater tailoring of these ATs to the individual [4,5,6,16,17,18,19]. On the other hand, this centering must take into account the development disparities of the various countries and the relative peculiarities of their living environments, particularly taking into account that development models based on high technology and costs are ill-suited to poor countries [22]. The UN with its operational branches, the WHO and UNICEF, is taking various actions in this area to improve the diffusion of ATs and monitor their progress. The actions of the WHO and UNICEF are in some respects synergistic and complementary. The WHO [1] has identified five *challenges* in the field of ATs (*the policy, the products, the provision, and the assistive technology within universal health coverage*), for which it is taking international action. In line with these and with the WHA71.8 [27] resolution, it has set up an observatory [26] in this area and has activated continuous monitoring at the population and system levels through two dedicated tools [28,29]. The data and metadata *are disseminated and updated* online and currently cover a portion of the population and governments. *Another level of scientific dissemination* by the WHO consists of the online publication of contents and guidelines through specific volumes dedicated to key strategic issues related to ATs. Three have been identified (all accessible online) that together address the subject of priority ATs at reasonable prices, the process and development specifications, and third-party supply through tenders [34,35,36].

UNICEF has also been active in specific initiatives in collaboration with the WHO. These initiatives concerned, for example, the introduction of 24 new products to the catalog, the indication and guidelines for specifications and essential requirements for the development of 26 priority products, and the global awareness of ATs through dissemination activities and workshops [24,25,26,27,28]. 

Of great scope and impact is the “Global Report on Assistive Technology” developed by UNICEF and WHO [29]. The report on ATs gives a strategic vision [42,63,64] for international access to ATs, with a focus on several issues, including the need and the expected economic benefits. Among the most important outputs of the document are 10 recommendations. Table 2 shows the opportunities that emerged from the analysis. 

#### 4.1.2. The Contribution of the Umbrella Review

In addition to the direct analysis of the contents of the institutional sites of the UN, UNICEF, and the WHO, an analysis was carried out by means of an *umbrella review* [62,65] of the systematic reviews produced in this area, which represent, as is known, the highest level of publications for the formation of evidence-based medicine.

This analysis has revealed a growth in interest in this theme in the recent period. The most relevant issues that have been addressed, including the effectiveness and usage, are the importance of the internet and EAT in ATs [44,45,53,55,60], AT technologies for cognitive and communication disabilities [47,49,52,54,56,58,61], and for motor disability [46,50]. An important space has been dedicated to the ICF, in line with what has been shown by direct research. The ICF comes to the fore both when issues of interconnection of ATs with the environment and activities are dealt with [43,48,57], and as a formal approach methodology [49,61]. Little space was given, for example, to the relevant issue of unequal access. Only one review addressed the limited access to ATs in LMIC [59]. The findings revealed there were limited AT options to prevent impairment and disability among older adults without current disabilities in the investigated countries (Brazil, Cambodia, Egypt, India, Turkey, and Zimbabwe). The study emphasized the need for a comprehensive, integrated approach within the health and social systems of LMICs to increase the availability of ATs for aging populations, promote low-cost AT initiatives, raise awareness, and build the capacity of ATs.

The literature overview highlights how consolidated scientific topics are influenced more by an interest in devices and their environment of use, passing through the importance of electronics and the internet. Support for raising awareness of access issues is a topic that has less extensive coverage. Now, the *umbrella review* does not report systematic reviews dedicated to monitoring initiatives at the three levels of system, needs, and satisfaction for ATs. This could have two explanations. The first, more reasonable, is that not enough data have yet been accumulated for the development of these reviews given that the monitoring tools of the WHO have only been released in recent times. The second, less probable, is that there is less interest from authors on these issues. On the other hand, if we focus on recent scientific articles conducted by the WHO experts themselves, the need to echo this issue [66] and the need to study critical aspects of the diffusion of the culture of ATs in some nations (which is also reflected in the participation in the surveys themselves carried out by the WHO and UNICEF) is particularly emphasized [65]. Furthermore, the importance and central role of ATs with regard to the realization of the Convention on the Rights of Persons with Disabilities [63] and the central role of monitoring tools are highlighted in the recent scientific contributions conducted by the WHO experts, both at the system level and at the need level of the ATs made available by the WHO [64,66,67].

Overall, the *umbrella review* shows an imperfect overlap of the domain of interventions of the ATs on the WHO/UNICEF/UN websites and in the scientific literature referring to these international bodies. This is partly due to the different roles of the contributors: the former have a more institutional role while the latter have a key role in development and research. Surely, actions linking the two sectors could be useful to enhance AT initiatives on issues dear to the WHO/UNICEF/UN.

### 4.2. Final Thought: The Bottlenecks

Surely, the actions of the considered institutions at the international level are multifaceted and of an important level of concreteness; think of the observatory [26] and the Global Report on ATs [42]. 

However, it must be borne in mind that the work ahead of us is considerable. For example, 70 out of 193 countries provided responses on the progress indicators tool, and not all of these (62) have specific legislation (see the data and metadata available online at [26]). However, in the administration of these tools, there is always a need for collaboration on the part of governments, and presumably, the neediest populations find themselves with governments with less evolved legislation and less collaboration toward these initiatives.

Greater collaboration between national and international institutions and between themselves regarding the dissemination of research activities could also be of great importance in tackling the hot topics in the AT field as they emerged in the first part of the overview of international sites. Table 3 shows the problems and bottlenecks that emerged from the analysis. 

## 5. Conclusions

In conclusion, it should be noted that important initiatives have been undertaken in the AT field by the United Nations through its institutional operational arms, the WHO and UNICEF. These initiatives range from the set-up of an observatory, the improvement of catalogs, the design of guidelines, and the dissemination of the culture on ATs, up to the design and submission of survey tools (both at the population and at the government level) and the planning of new ones. From a general point of view, the overview conducted on the websites of the WHO, UN, and UNICEF highlighted that the initiatives are concrete and potentially have a high impact. However, there are also bottlenecks. Information campaigns on ATs are very important, but the effectiveness must also be monitored through specific targeted scientific investigations. In this, scholars have an important role. Indeed, not all countries are responding to these actions; the neediest populations, often with governments with less evolved legislation, seem to be less collaborative towards these initiatives. From another point of view, an *umbrella review* highlighted how consolidated scientific topics are influenced more by an interest in devices and their environment of use and digitalization. The support for raising awareness of access to ATs has less extensive coverage as a theme of interest. The need to echo the issue along with the need to study critical aspects of the diffusion of the culture of ATs in some national systems (which is also reflected in the participation in the surveys themselves carried out by the WHO and UNICEF) is particularly remarked in very recent targeted studies. All of this highlights the need to better bring together the institutional world (national and international) with the world of dissemination of the scientific literature.

## Figures and Tables

**Table 1:** The recommendations from the Global Report on Assistive Technology.

Recommendation #	Description
*1*	Improve access to assistive technology within all key development sectors.
*2*	Ensure that assistive products are safe, effective, and affordable.
*3*	Enlarge, diversify, and improve human resource capacity.
*4*	Actively involve users of assistive technology and their families.
*5*	Increase public awareness, garner political support, and combat stigma surrounding assistive technology use.
*6*	Invest in data- and evidence-based policy.
*7*	Invest in research, innovation, and an enabling ecosystem.
*8*	Develop and invest in enabling environments.
*9*	Include assistive technology in humanitarian responses.
*10*	Provide technical and economic assistance through international cooperation to support national efforts.

**Table 2:** Opportunities emerged from the analysis.

#	Opportunities
*1*	Increased awareness of and interest in assistive technology devices due to the pandemic.
*2*	Greater focus on tailoring assistive technology to the individual needs of people with disabilities.
*3*	International bodies such as the UN, WHO, and UNICEF working to improve the diffusion of assistive technology devices and monitoring their progress.
*4*	Online dissemination of guidelines and contents related to assistive technology devices.
*5*	Introduction of new products in catalogs and global awareness through dissemination activities and workshops.
*6*	The Global Report on Assistive Technology providing a strategic vision of international access to assistive technology and economic benefits.
*7*	Collaborative work among governments, institutions, and population.

**Table 3:** Problems/bottlenecks emerged from the analysis.

#	Problems/Bottlenecks
*1*	Population growth and gradual aging lead to a growth in people with disabilities who need support and assistance.
*2*	Development disparities of various countries and the relative peculiarities of living environments affect the adoption of assistive technology devices.
*3*	High technology and costs may not be suitable for poor countries.
*4*	Lack of specific legislation in some countries hinders the progress and adoption of assistive technology devices.
*5*	Need of collaboration from governments, particularly those with less-evolved legislation and less collaboration toward initiatives.
